# Dendritic Forest-Like Ag Nanostructures Prepared Using Fluoride-Assisted Galvanic Replacement Reaction for SERS Applications

**DOI:** 10.3390/nano11061359

**Published:** 2021-05-21

**Authors:** Ming-Hua Shiao, Tsunghsueh Wu, Hung Ji Huang, Ching-Yi Peng, Yung-Sheng Lin, Ting-Yu Lai, Yang-Wei Lin

**Affiliations:** 1 Taiwan Instrument Research Institute, National Applied Research Laboratories, Hsinchu 300092, Taiwan; mhshiao@tiri.narl.org.tw (M.-H.S.); hjhuang@narlabs.org.tw (H.J.H.); 2 Department of Chemistry, University of Wisconsin-Platteville, 1 University Plaza, Platteville, WI 53818-3099, USA; wut@uwplatt.edu; 3 Department of Chemical Engineering, National United University, Miaoli 360001, Taiwan; s7614693@gmail.com; 4 Department of Chemistry, National Changhua University of Education, Changhua City 50007, Taiwan; superhuman860731@gmail.com

**Keywords:** dendritic forest-like Ag nanostructures, fluoride-assisted galvanic replacement reaction synthesis, surface-enhanced Raman scattering spectroscopy

## Abstract

Dendritic forest-like Ag nanostructures were deposited on a silicon wafer through fluoride-assisted galvanic replacement reaction (FAGRR) in aqueous AgNO_3_ and buffered oxide etchant. The prepared nanostructures were characterized using scanning electron microscopy, energy-dispersive X-ray spectroscopy, inductively coupled plasma–optical emission spectroscopy, a surface profiler (alpha step), and X-ray diffraction. Additionally, the dendritic forest-like Ag nanostructures were characterized using surface-enhanced Raman scattering (SERS) when a 4-mercaptobenzoic acid (4-MBA) monolayer was adsorbed on the Ag surface. The Ag nanostructures exhibited intense SERS signal from 4-MBA because of their rough surface, and this intense signal led to an intense local electromagnetic field upon electromagnetic excitation. The enhancement factor for 4-MBA molecules adsorbed on the Ag nanostructures was calculated to be 9.18 × 10^8^. Furthermore, common Raman reporters such as rhodamine 6G, 4-aminothiolphenol, 5,5′-dithiobis-2-nitrobenzoic acid, and carboxyfluorescein (FAM) were characterized on these dendritic forest-like Ag nanostructures, leading to the development of an ultrasensitive SERS-based DNA sensor with a limit of detection of 33.5 nM of 15-mer oligonucleotide.

## 1. Introduction

Surface-enhanced Raman scattering (SERS) is well known for its high sensitivity toward analytes that adsorb on roughened Au and Ag surfaces [1]. Studies have discovered that electromagnetic fields and chemical amelioration enhance the Raman signals of molecules adsorbed on metallic nanostructures with a factor of 10^2^ to 10^6^. Thus, the limits of detection (LODs) in the range 10^−9^ to 10^−12^ M have been achieved, making SERS a highly sensitive technique for use in biochemical sensing, medical diagnostics, and environmental monitoring [2,3]. Colloidal Ag nanomaterials of various morphology have been widely investigated as highly efficient SERS substrates [4,5,6]. The anisotropic growth of Ag microstructures is particularly attractive because these microstructures possess a rough surface that induces localized surface plasmon resonance when they interact with light and thus strongly enhance the electromagnetic field in SERS [7,8,9].

High-quality anisotropic Ag nanostructures can be obtained through many methods, including photolithography and template growth from electrodeposition, in which the surface roughness of Ag nanostructure can be precisely controlled during fabrication to yield a greatly enhanced SERS signal for highly sensitive detection of brilliant cresyl blue, benzenethiol, and adenine [10,11,12,13,14,15,16]. Both photolithography and template growth from electrodeposition require sophisticated surface patterning steps and a time-consuming bottom-up approach to build nanostructures on the substrate. Since these two methods were first reported, researchers have attempted to develop simple and creative approaches for generating anisotropic Ag nanostructures. For example, Yang et al. used enzyme catalytic reaction to prepared SERS-active Au@Ag nanostructure. First, alkaline phosphatase (ALP) dephosphorylated sodium L-ascorbyl-2-phosphate to form ascorbate. At the same time, Ag^+^ ions could be reduced to Ag atoms which deposited onto the Au surface. However, this method has the drawback of using expensive ALP enzyme [17]. Flower-like Ag seed formed through self-assembly was employed as a template for the formation of flower-like Ag@molecularly imprinted polymers (MIPs) nanostructures. This flower-like Ag@MIPs nanostructures used as a SERS substrate provided the high sensitive detection of glibenclamide with the detection of limit of 1 ng mL^−1^ [9]. However, this method requires a template for synthesis. To further simplify SERS substrate production, Lin’s group demonstrated a relatively easy, inexpensive, and template-free synthetic route for directly electrodepositing SERS-active flower-like Ag mesoparticles on a screen-printed carbon electrode (SPCE) substrate through electrochemical method [18,19]. The enhancement factor (EF) of 4-mercaptobenzoic acid (4-MBA) molecules on the flower-like Ag mesoparticle surface was approximately to be 1.3 × 10^5^. As the development of SERS substrates continues, a simple method that focuses on the preparation of anisotropic Ag structures is desirable.

In this study, dendritic forest-like Ag nanostructures were deposited on silicon wafers through a fluoride-assisted galvanic replacement reaction (FAGRR) involving aqueous AgNO_3_ and buffered oxide etchant [20,21]. FAGRR involves the following steps: F^−^ ions in aqueous AgNO_3_ and buffered oxide etchant oxidize Si atoms, producing SiF_6_^2−^ and releasing four electrons. The generated electrons flow to suitable positions on the surface of Si or Ag, where Ag^+^ is reduced to Ag. The growth of Ag nanoparticles thus depends on the Ag crystal structure, electrons’ conductivity in Ag and the Si substrate, Ag^+^ diffusion in the solution, and the reduction process. The Ag nanostructures have a rough surface and interlaced structure ideal for generating SERS hot spots with high sensitivity. Therefore, these Ag nanostructures provide an intense SERS signal from adsorbed 4-MBA acting as a Raman reporter. The EF of 4-MBA molecules on the dendritic forest-like Ag nanostructures was experimentally determined. The signal reproducibility of the prepared Ag nanostructures was investigated. For practicality, the prepared dendritic forest-like Ag nanostructures were used to detect single-stranded DNA through sequence-selective hybridization.

## 2. Materials and Methods

### 2.1. Materials

Chemicals—AgNO_3_, buffered oxide etchant (BOE), acetone, methanol, dimethyl sulfoxide (DMSO), 4-MBA, 5,5′-dithiobis-2-nitrobenzoic acid (DTNB), Rhodamine 6G (R6G), and 4-aminothiolphenol (4-ATP)—were of spectrophotometry-grade and obtained from Sigma-Aldrich (Milwaukee, WI, USA). BOE is a mixture of a buffering agent solution prepared by NH_4_F and HF. For simply, the mixture of 11.4% NH_4_F and 2.3% HF (*v*/*v*%) represented as 1.0× BOE. Single-strand DNA sequences (probe: 5′-CCAGATACTCACCGG-SH-3′, target: 5′-FAM-CCGGTGAGTATCTGG-3′, random: 5′-FAM-ACCGTAAGTACCCGC-3′) were bought from Genomic (New Taipei City, Taiwan). N-type silicon wafer was obtained from the Summit-Tech Company (Hsinchu, Taiwan). Deionized water (Milli-Q ultrapure system, Millipore, MA, USA) was used throughout the study.

### 2.2. Fabrication of Dendritic Forest-Like Ag Nanostructures on a Si Wafer

Dendritic forest-like Ag nanostructures were deposited on Si wafer using the FAGRR method. In this study, synthesis of the dendritic forest-like Ag nanostructures began with cleaning a 2 × 2 cm^2^ n-type Si wafer through ultrasonic washing with acetone, methanol, and deionized water for 5 min. The substrate was dried using N_2_ spray for 5 min and baked at 120 °C in a covered glass Petri dish in an oven for 5 min. The native oxide layer on the Si substrate was removed by soaking the substrate in 10% HF solution for 10 s; this step was crucial because HF etching increased the roughness of the Si substrate and adhesion of the synthesized Ag nanotrees. The Si wafer was treated in a mixture comprising 24 mL of the reactant solution (0.02–0.10 M AgNO_3_ and 0.083–1.0× BOE solution) for different durations (0.5–10 min) in a Teflon container measuring 5 × 5 × 5 cm^3^. The synthesized dendritic forest-like Ag nanostructures were washed two or three times with deionized water. The Ag nanostructures deposited on the Si wafer were dried with N_2_ spray and then incubated at 120 °C for 5 min.

### 2.3. Characterization

The size and shape of the Ag nanostructures were observed using scanning electron microscopy (SEM, SU-8010, Hitachi, Tokyo, Japan), and the composition of the structures was determined through energy-dispersive X-ray spectroscopy (EDS, SU-8010, Hitachi, Tokyo, Japan). Surface morphology of the dendritic forest-like Ag nanostructures was intensively investigated by an atomic force microscope (AFM, Digital Instruments Dimension 3100, Veeco Instruments Inc., Santa Barbara, CA, USA) in tapping mode where scanned areas were set to (1 × 1) micrometer. Debris were carefully avoided in order to obtain high resolution clear images for further roughness analysis. The roughness of the prepared dendritic forest-like Ag nanostructures was evaluated using a surface profiler (XP-2, Ambios Technologies, Santa Cruz, CA, USA). With constant pressure and moving rate, the diamond tip of surface profilomer was scanning cross the surface of specimen to obtain the surface roughness. X-ray diffraction (XRD) was achieved using a diffractometer (D8 Discover, Bruker, Billerica, MA, USA) with Cu Kα radiation (λ = 0.15418 nm). High-resolution inductively coupled plasma (ICP) mass spectrometry (Element 2, Thermo Fisher Scientific, Waltham, MA, USA) was used to measure the weight of Ag nanostructures deposited on a Si wafer.

### 2.4. SERS Analysis Procedure

For optimal combination of SERS measurements, the prepared Ag substrates were totally immersed into certain concentration of 4-MBA (1 × 10^−4^ M, DMSO was used as solvent) under dark atmosphere for 8 h and then dried spontaneously. In order to evaluate EF, the clean silicon wafers and optimal combination Ag substrates were also totally immersed into 10^−2^ M and 5 × 10^−10^ M 4-MBA, respectively, under dark circumstance for 8 h and then dried spontaneously. For the practicality of the prepared Ag substrate, common Raman reporters for SERS sensors were tested including R6G, 4-ATP, DTNB, and FAM molecules. The prepared Ag substrates were immersed in ethanolic R6G (20 nM), 4-ATP (1.4 µM), DTNB (100 µM), and FAM (1.0 µM) under dark atmosphere for 8 h and then dried spontaneously. The SERS measurements were performed using a confocal micro-Raman system (Thermo Scientific Inc., New York, NY, USA) with 532-nm laser beam as an excitation source for SERS analysis. The exciting laser power was about 2 mW and the acquisition time used for each spectrum was 10 s. Raman scattering signals were collected by using a 10× objective lens (numerical aperture of 0.25) and then detected using a spectrometer.

### 2.5. DNA Probe Modification and Target Hybridization

For DNA probe modified on the surface of dendritic forest-like Ag nanostructures, 20 µL of DNA probe (5′-CCAGATACTCACCGG-SH-3′, 10 µM) was dissolved in 2 mL of phosphate buffer (pH 7.2) and clean forest-like Ag substrate was immersed in this solution at 25 °C for 12 h. Through strong Ag-S bonding, DNA modified Ag substrate was washed by phosphate buffer, deionized water and dried. For the hybridization experiments, DNA modified Ag substrates in the annealing solution (5 mL, 10 mM phosphate buffer, pH 7.2, 50 mM NaCl) containing different concentrations of target DAN (5′-FAM-CCGGTGAGTATCTGG-3′) were heated to 95 °C for 3 min and gradually cooled to 25 °C. Afterward, the Ag substrate was washed by annealing buffer, deionized water and dried at ambient conditions for SERS analysis.

## 3. Results and Discussion

### 3.1. Characterization of Dendritic Forest-Like Ag Nanostructures

**Figure 1:**
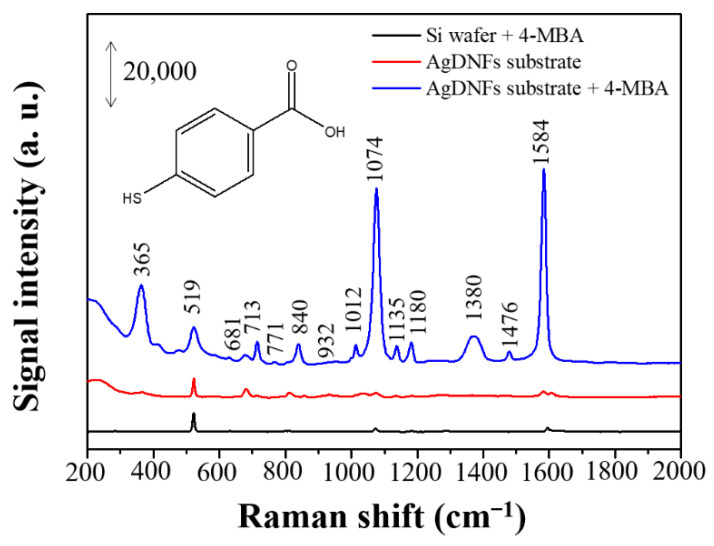
Normal Raman spectrum of 10 mM 4-MBA (black line) on silicon wafer and SERS spectra of 0 µM (red line) and 100 µM 4-MBA (blue line) on the dendritic forest-like Ag nanostructures.

SERS is a powerful analytical tool for the quantitative analysis of target analytes in the fields of biochemical sensing, medical diagnostics, and environmental monitoring. The surface morphology and nanostructure of the substrate crucially influence the degree of Raman signal enhancement in SERS, and the dendritic forest-like Ag nanostructures synthesized in this study had distinctive morphology and a rough surface, making them as adequate SERS-active substrates. To explore their SERS performance, 4-MBA was used as a Raman reporter because it is facile to form a self-assembled monolayer on the Au or Ag surface and has been thoroughly investigated using SERS [22,23]. Figure 1 represents the SERS spectra of 4-MBA on the dendritic forest-like Ag nanostructures. An intense Raman signal (in blue line) was obtained from the 4-MBA monolayer on the Ag nanostructures, whereas weak signal regarded as background (in red line) was obtained in the absence of a 4-MBA monolayer. Although others peaks are visible at higher Raman shifts despite having low intensities (in red line), they maybe to contaminants resulting from the manufacturing process of the dendritic forest-like Ag nanostructures. Furthermore, at the optimal combination of SERS procedure, this background signal can be considered negligible after adding the 4-MBA. The Raman peaks in Figure 1 were found at 365, 519, 681, 713, 771, 840, 932, 1012, 1074, 1135, 1180, 1380, 1476, and 1584 cm^−1^. The observed vibration modes are shown in Table 1 [23,24,25,26,27]. The Raman peaks at 1584 and 1476 cm^−1^ were assigned to 9a and 15, respectively. The Raman peak at 1380 cm^−1^ was attributed from the O–H bending, the carboxyl carbon and aromatic carbon stretching, 19a coupled with CO_2_ asymmetric stretching. The Raman peaks at 1180 and 1135 cm^−1^ were assigned to 9a coupled with O–H bending and rocking, respectively. The Raman peaks at 1074 and 1012 cm^−1^ were assigned to 1 coupled with C–S and C–O stretching, respectively. The Raman peaks at 932 and 840 cm^−1^ arose from 17a and 10b, respectively. The Raman peak at 771 cm^−1^ was attributed from 6a coupled with the carboxyl carbon and aromatic carbon stretching and the C–O stretching. The peak at 713 cm^−1^ arises from 11 coupled with CO_2_ wagging. The Raman peak at 681 cm^−1^ arises form CO_2_ bending, 19a coupled with C–S stretching. In addition, normal Raman spectrum of 4-MBA on silicon wafer (in black line) was also found. Due to lacking a strong electric field enhancement by Ag nanostructures, weak signal was obtained. This normal Raman spectrum can be used for calculating Raman EF in the later section. Although the peak positions and intensities of the Raman spectra would be varied by different substrate, most of the Raman peaks are similar [26]. Additionally, the Raman peak at 519 cm^−1^ can be found in all spectra (in black, red and blue line) and arises from the silicon wafer. However, the reason why different peak width is still studied in the laboratory.

**Figure 2:**
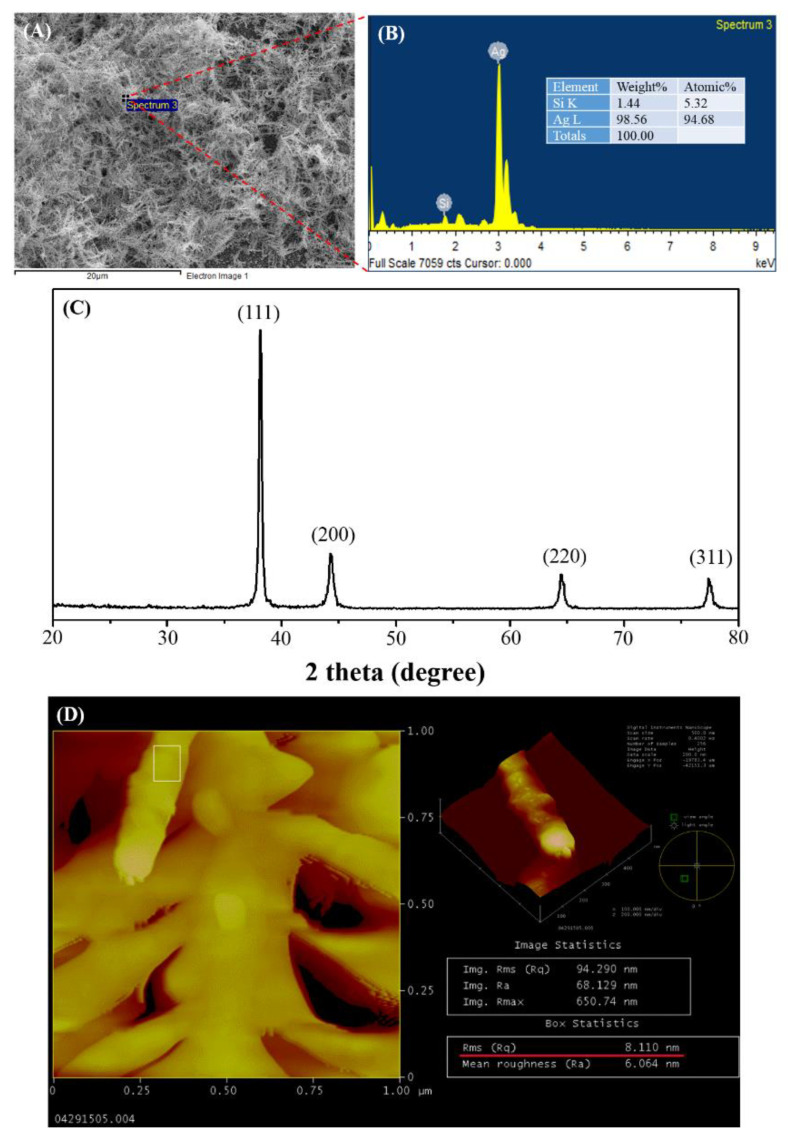
(**A**) SEM image, (**B**) EDS spectrum, (**C**) XRD spectrum, and (**D**) AFM image of the dendritic forest-like Ag nanostructures on a Si wafer.

The surface morphology of dendritic forest-like Ag nanostructures on the smooth Si wafer was well-dispersed, thick, and dendritic forest-like structures with size smaller than 100 nm, as shown in the SEM image in Figure 2A. The Ag nanostructures consisted of sharp spikes, which can act as an amplifier of the electromagnetic field when interacting with light to generate local surface plasmonic waves and thus intensified Raman signals. Next, the elemental composition of the dendritic forest-like Ag nanostructures was confirmed using EDS and XRD, which revealed the existence of Ag atoms (Figure 2B,C, respectively). The diffraction peaks at 38.1°, 41.2°, and 64.5°, corresponding to (111), (200), and (220) crystal planes of face-centered-cubic Ag (JCPDS 65-2871), were also found, indicating the existence of polycrystalline Ag. Figure 2D represented AFM images for the prepared dendritic forest-like Ag nanostructures. The result indicated that the branch of the prepared dendritic forest-like Ag nanostructures possessed relatively high roughness (8.11 nm) comparing to the polished silicon wafer (~0.3 nm).

### 3.2. Optimal Combination of SERS Performance

**Figure 3:**
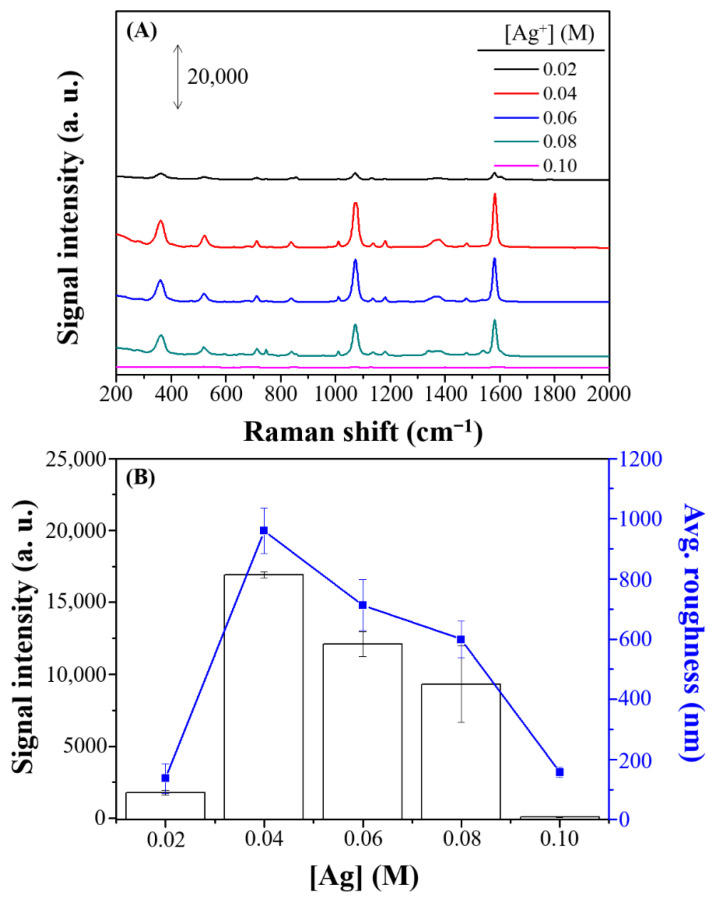
(**A**) SERS spectra and (**B**) Raman intensity and average roughness (nm) of 100 µM 4-MBA on Ag nanostructures prepared with various concentrations of Ag^+^ ions (0.02, 0.04, 0.06, 0.08, and 0.10 M). The error bars represent the standard deviations by using three batches of Ag substrates for which the average signal of 10 spots per sample are recorded.

Factors such as concentration of Ag^+^ for crystal growth, etching effect, and reaction time were identified as potentially influencing Raman enhancement and were thus investigated to optimize the experimental protocol for this newly developed SERS substrate. First, concentrations of Ag^+^ ions ranging from 0.02 to 0.10 M (Figure 3A) were evaluated, and the Raman intensity of 4-MBA signal peaked at 0.04 M Ag^+^ (white block in Figure 3B). This suggested that the surface morphology and roughness of the Ag structures varied with Ag^+^ concentration. Thus, the surface morphology and average roughness of different Ag structures were investigated further using SEM and a surface profiler. Blue line in Figure 3B represented the surface roughness of the prepared Ag substrate by using a α-step surface profilometer. The tendency of the roughness for the prepared Ag substrates was similar to that of the Raman intensity.

**Figure 4:**
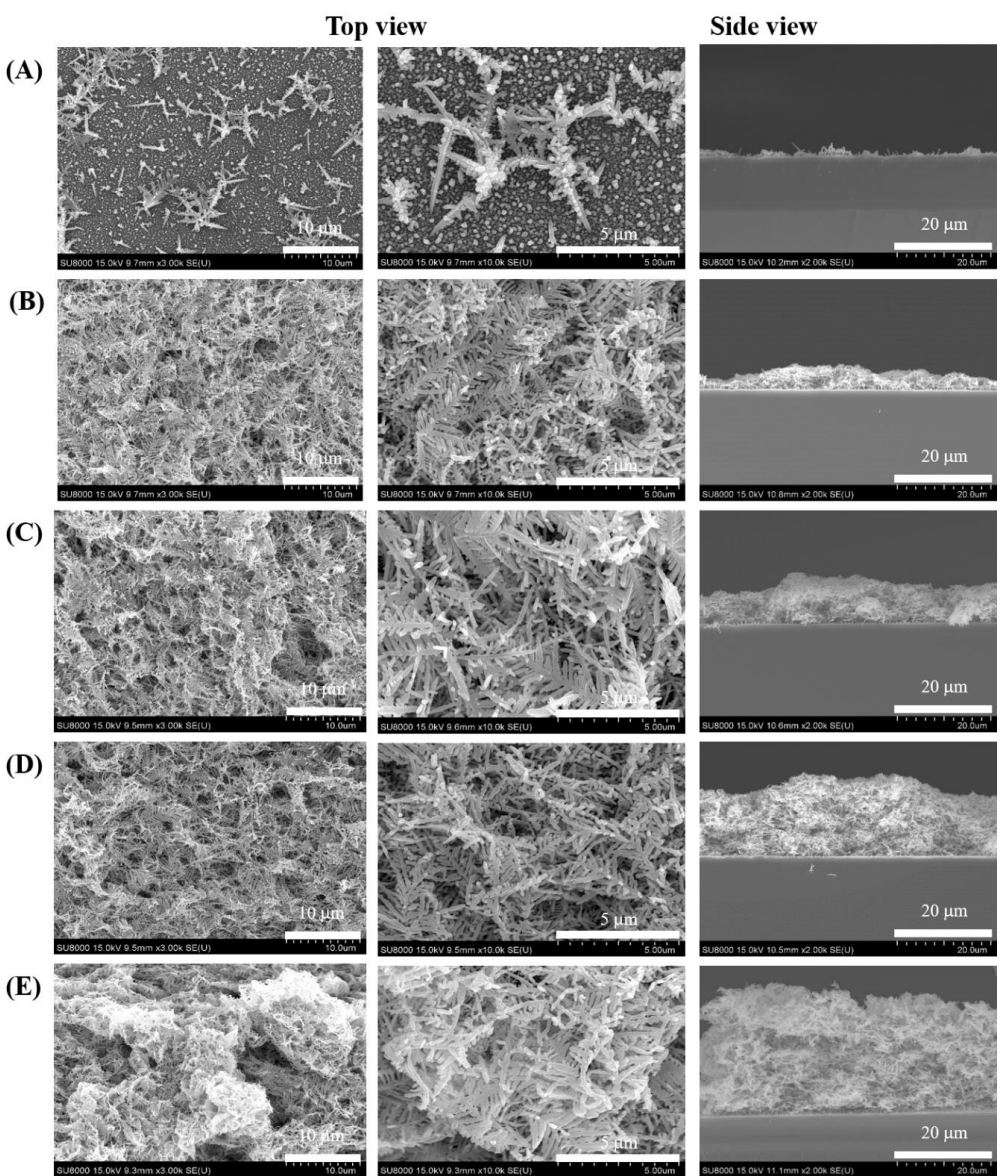
Representative SEM images of the Ag nanostructures prepared at various Ag^+^ ion concentrations: (**A**) 0.02, (**B**) 0.04, (**C**) 0.06, (**D**) 0.08, and (**E**) 0.10 M. Scale bar: 10 µm, 5 µm (top view) and 20 µm (side view).

As shown in the side-view SEM image, the height of Ag deposits increased with an increase in Ag^+^ ion concentration; the top-view SEM image indicates that the Ag deposits were denser when the concentration was higher (Figure 4). Greater deposit height and density both reduce the amount of excitation accessible to 4-MBA molecules adsorbed on the inner layer of the Ag nanostructures, leading to lower Raman intensity. At Ag concentrations higher than 0.06 M, the deposition mode known as Ostwald ripening favored smoother features (Figure 4 in top view), significantly reducing both the surface roughness and Raman intensity. Thus, the optimal concentration of Ag^+^ ions was determined to be 0.04 M for deposition of Ag nanostructures on a Si wafer.

**Figure 5:**
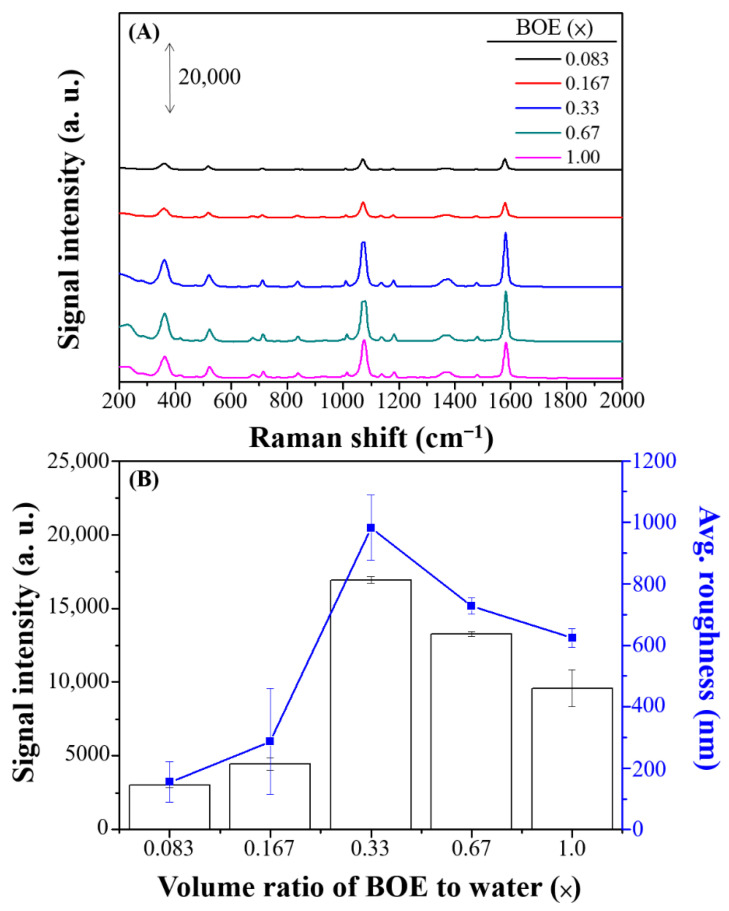
(**A**) SERS spectra and (**B**) Raman intensity and average roughness (nm) of 100 µM 4-MBA on Ag nanostructures prepared with various volume ratios of BOE to deionized water (0.083×, 0.167×, 0.33×, 0.67×, and 1.00×). The error bars represent the standard deviations by using three batches of Ag substrates for which the average signal of 10 spots per sample are recorded.

The etching effect of the BOE was investigated by collecting the SERS spectra of 4-MBA at volume ratios of BOE to deionized water (total volume: 24 mL) ranging from 0.083× to 1.0× (Figure 5A). The maximum Raman intensity of 4-MBA was found when the BOE concentration was 0.33×, as illustrated in Figure 5B. At a higher volume ratio, the flux of electrons was higher due to the high concentration of F^−^ ions interacting with the Si wafer, leading to rapid formation of a larger Ag base.

**Figure 6:**
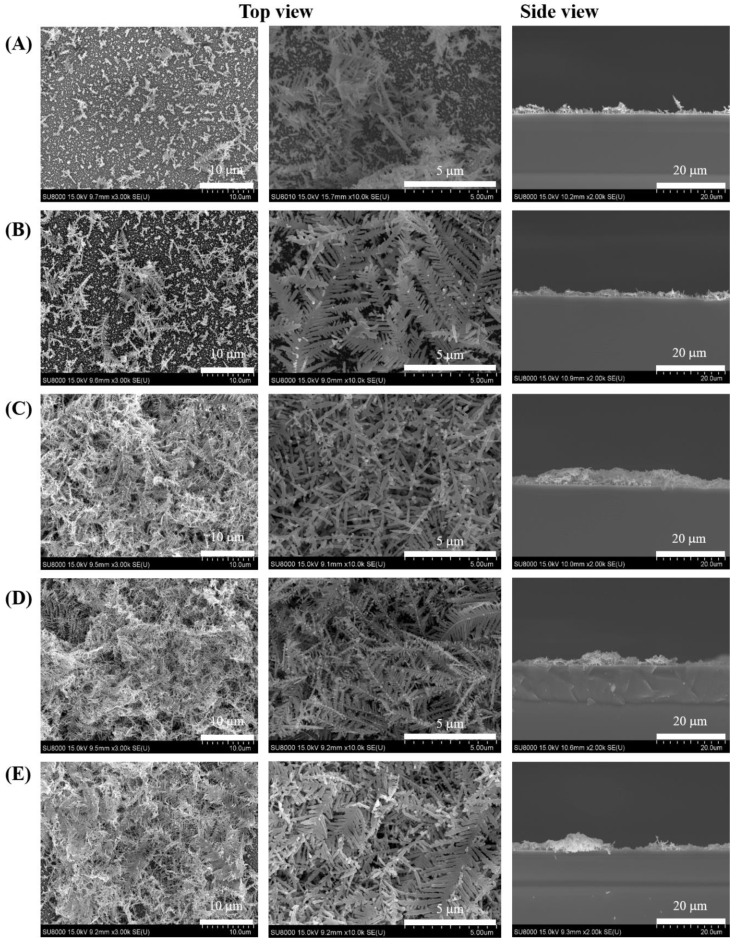
Representative SEM images of the Ag nanostructures prepared with various volume ratios of BOE to deionized water: (**A**) 0.083, (**B**) 0.167, (**C**) 0.33, (**D**) 0.67, and (**E**) 1.00×. Scale bar: 10 µm, 5 µm (top view) and 20 µm (side view).

Through the repeated studies and SEM profiling presented in Figure 6, this study revealed that a larger Ag base adhered less strongly to the Si wafer. Thus, the surface area of the 4-MBA adsorbed on the Ag surface was lower, and the Raman intensity was considerably lower. Figure 6 presents images of the surface of the prepared Ag substrate from the top view and reveals the coverage. The surface profiler results (blue line in Figure 5B) indicated that the maximum roughness was obtained at 0.33× BOE, which was consistent with the signal tendency. Therefore, the BOE volume ratio of 0.33× was applied in subsequent experiments.

**Figure 7:**
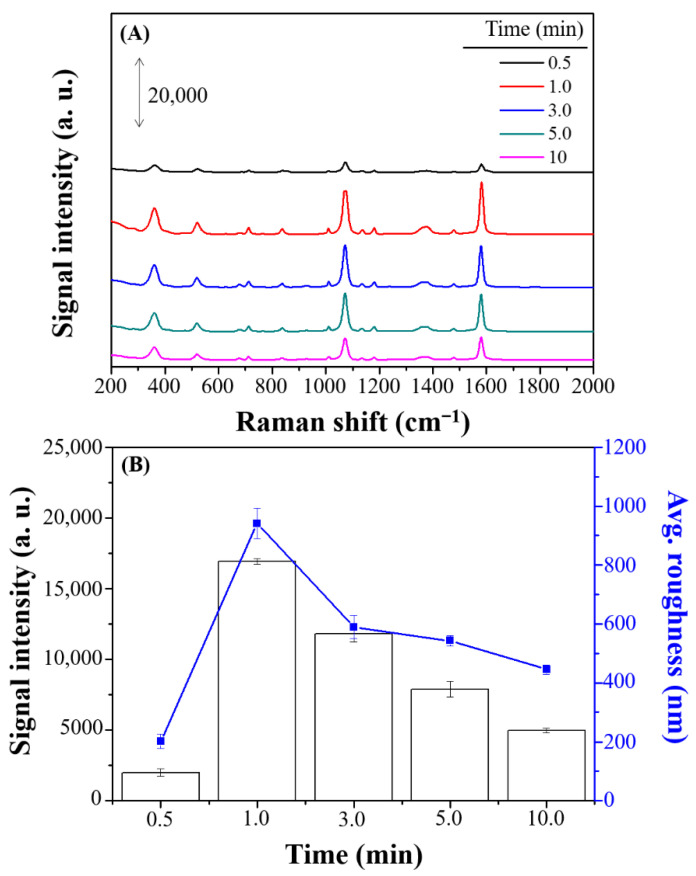
(**A**) SERS spectra and (**B**) Raman intensity and average roughness (nm) of 100 µM 4-MBA on Ag nanostructures prepared with various reaction times (0.5, 1.0, 3.0, 5.0, and 10 min). The error bars represent the standard deviations by using three batches of Ag substrates for which the average signal of 10 spots per sample are recorded.

**Figure 8:**
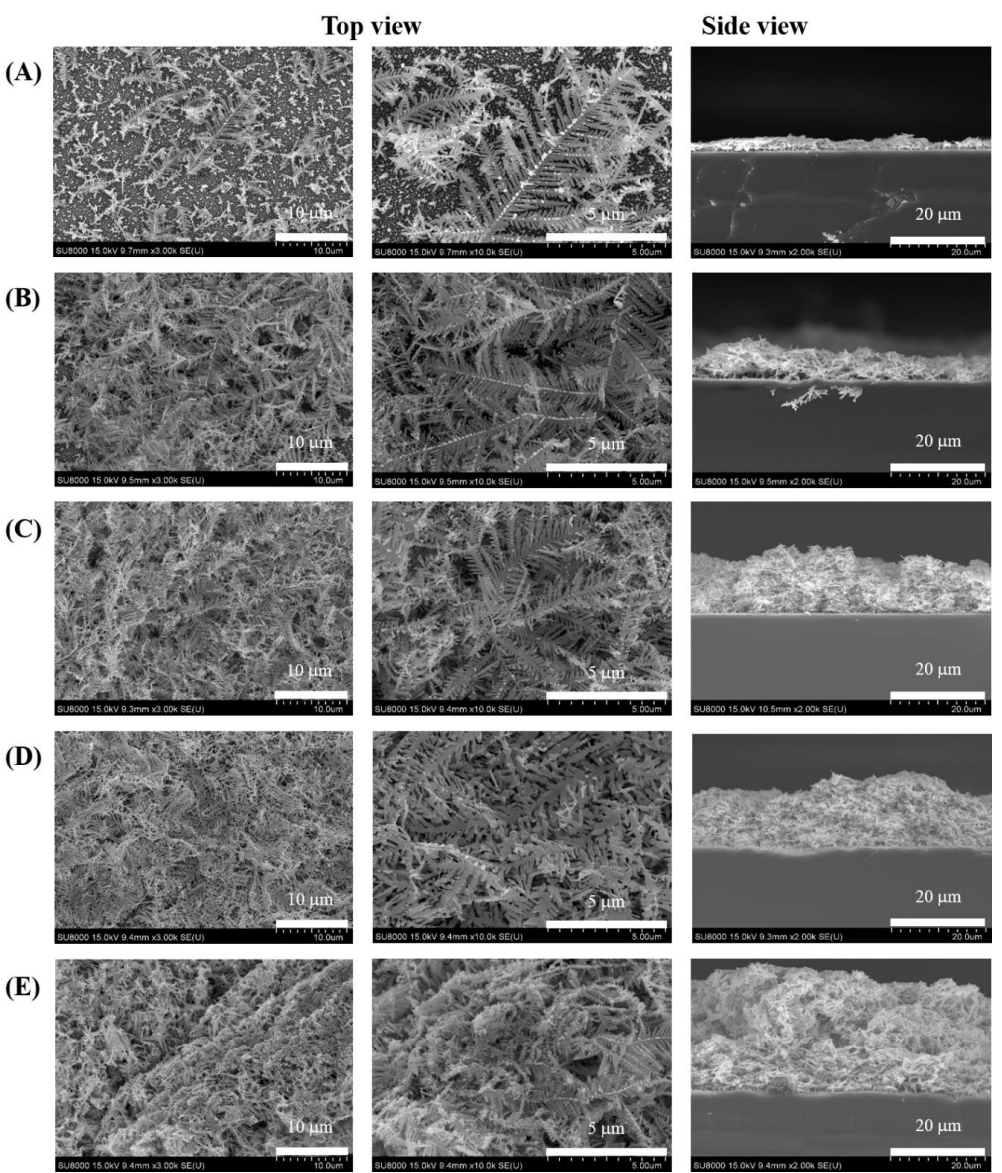
Representative SEM images of Ag nanostructures prepared with various reaction times: (**A**) 0.5, (**B**) 1.0, (**C**) 3.0, (**D**) 5.0, and (**E**) 10.0 min. Scale bar: 10 µm, 5 µm (top view) and 20 µm (side view).

The influence of reaction time on the height of Ag deposition was investigated (Figure 7A). A reaction time longer than 1 min resulted in excessive growth of Ag structures, rendering the surface smoother and thicker (Figure 8) and resulting in a reduced Raman signal (Figure 7B). Thus, 1.0 min was selected as the optimal reaction time for further study.

After Ag deposition under the optimal conditions (0.04 M Ag^+^ in the deposition solution, 0.33× BOE, and 1-min reaction time), the Ag surface density was analyzed using ICP–optical emission spectroscopy, and the silver distribution was found to be 0.4425 mg⋅cm^−2^. Based on the Raman spectra at 1584 cm^−1^, the EF of 4-MBA molecules adsorbed on silicon wafer and as-prepared Ag substrate can be further estimated according the equation:

(1) EF=ISERSNSERSINORNNOR

where *I_SERS_* and *I_NOR_* are the Raman intensities of SERS and normal Raman spectrum of the same vibration peak for 4-MBA molecule, respectively. Additionally, *N_SERS_* and *N_NOR_* represent the corresponding number of 4-MBA molecules exposed to the laser spot focused area. Because the laser parameters adopted in the SERS measurement were the same, *N_SERS_* and *N_NOR_* can be approximately determined by the concentration of 4-MBA, this empirical calculation has been verified in many previous work [18,19,28,29,30]. According to this equation, the EF of SERS for 4-MBA molecules was estimated to be about 9.18 × 10^8^, indicating that the prepared dendritic forest-like Ag nanostructures can be regarded as SERS active substrates. According to our previous study, the prepared dendritic forest-like Ag nanostructures also had the plasmonic effect [31]. Plasmons represent the collective motion of free electrons and can also induce additional light absorption and light extraction. Light absorption results from light trapping, and multiple scattering to produce surface plasmon in the layer of deposited Ag nanostructures, thereby enhancing the generation of Raman scattering signals. Besides plasmonic effect, surface roughness and surface area of the prepared dendritic forest-like Ag nanostructures—which were also attributed to the greater electromagnetic field enhancement and number of adsorption sites, respectively—led to a dramatic increase in the SERS intensity and EF value. In addition, a similar-morphology-structure substrate was found by Ge and Chen [26], but optimized combination procedure was not performed in the study, as a result of a lower EF value was provided for their proposed substrate. Table 2 represents that fabrication method and EFs of various flower-like Ag substrates. Our proposed method possessed inexpensive, simple, short preparation time and excellent SERS enhancement.

**Figure 9:**
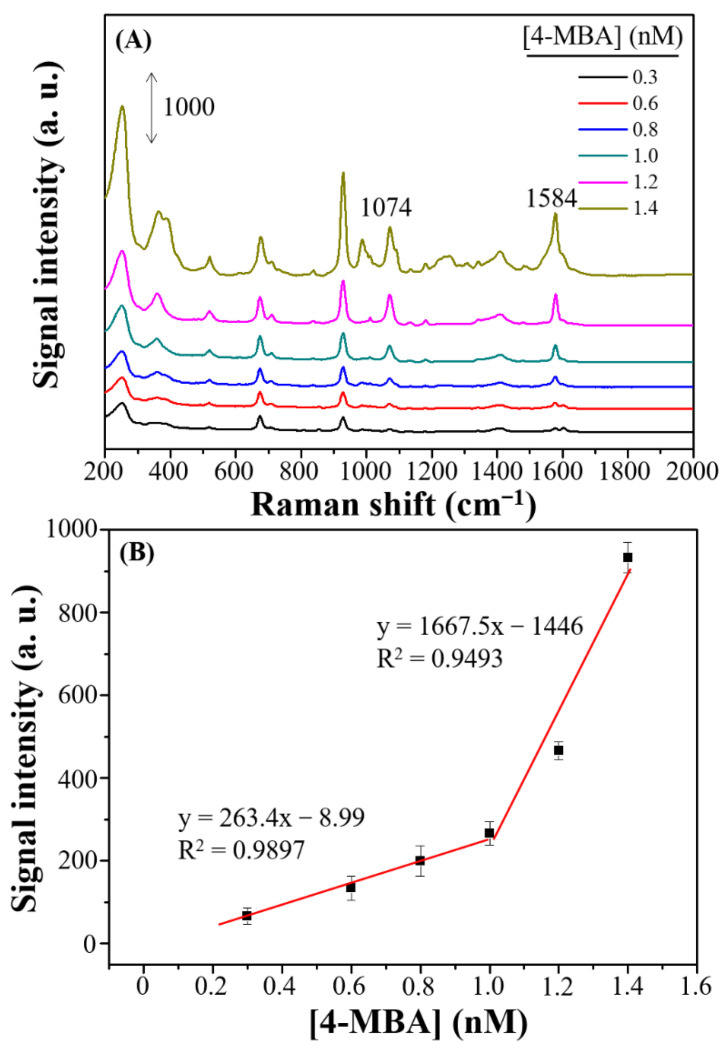
(**A**) SERS spectra at various 4-MBA concentrations on dendritic forest-like Ag nanostructures. (**B**) Linear responses of the Raman intensity plotted with respect to the concentration of 4-MBA. The error bars represent the standard deviations for triplicate experiments using three batches of Ag substrates.

Figure 9A shows the SERS spectra of self-assembled monolayers grown on the optimized Ag nanostructures on a Si wafer from a series of diluted 4-MBA solutions. The Raman shift at 1584 cm^−1^ caused by the 4-MBA molecules was obtained at a concentration as low as 0.3 nM. At this concentration, characteristic SERS peak at 1584 cm^−1^ still appeared clearly, however, when the concentration of 4-MBA was lower than 0.3 nM, no SERS peak at 1584 cm^−1^ was found. Thus, the limit of quantification (LOQ) for 4-MBA on the dendritic forest-like Ag nanostructures was 0.3 nM. Figure 9B indicated that the Raman signal at 1584 cm^−1^ was linear with respect to the 4-MBA concentration over the range 0.3 to 1.0 nM (R^2^ = 0.9897) and 1.0 to 1.4 nM (R^2^ = 0.9493), respectively.

### 3.3. Precision and Application of Dendritic Forest-Like Ag Nanostructures

**Figure 10:**
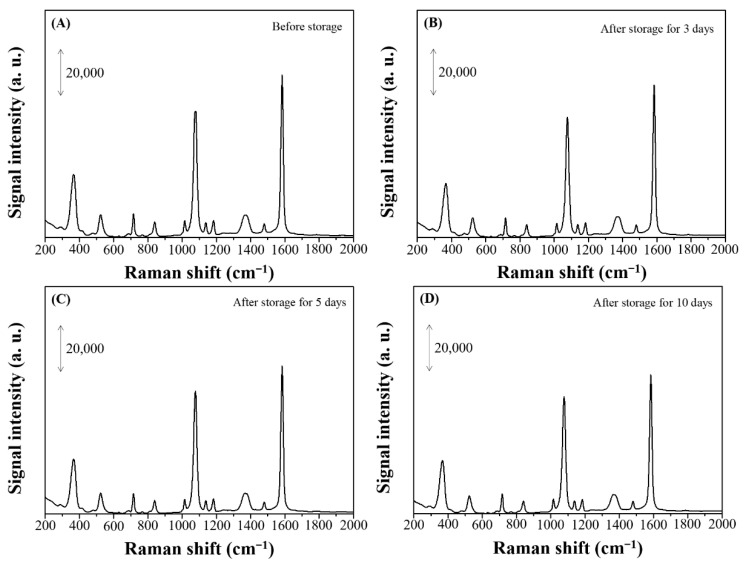
The SERS spectra of 4-MBA (100 µM) adsorbed on the dendritic forest-like Ag nanostructures (**A**) before and after storage for (**B**) 3, (**C**) 5 and (**D**) 10 days.

To test the stability of the dendritic forest-like Ag nanostructures as a SERS substrate, the intraday and interday reproducibility of the Raman intensity at 1074 and 1584 cm^−1^ was evaluated. Table 3 represents the Raman intensity of 4-MBA on different batches of dendritic forest-like Ag nanostructures and demonstrates the high SERS intensity, reproducibility, and stability of the nanostructures. Low-intensity fluctuations were attributed to variation during the adsorption of 4-MBA molecules (relative standard deviation [RSD%] < 0.618%). Regardless, the SERS intensity was relatively stable, demonstrating that this new SERS substrate is ideal because of the controllable FAGRR reaction. Moreover, the SERS spectra of 4-MBA (100 µM) adsorbed on the dendritic forest-like Ag nanostructures before and after storage for several days are shown in Figure 10. It shows that no significant change can be found in both the Raman shift and the intensity, where 84.5% SERS intensity can be maintained after storage for 10 days.

**Figure 11:**
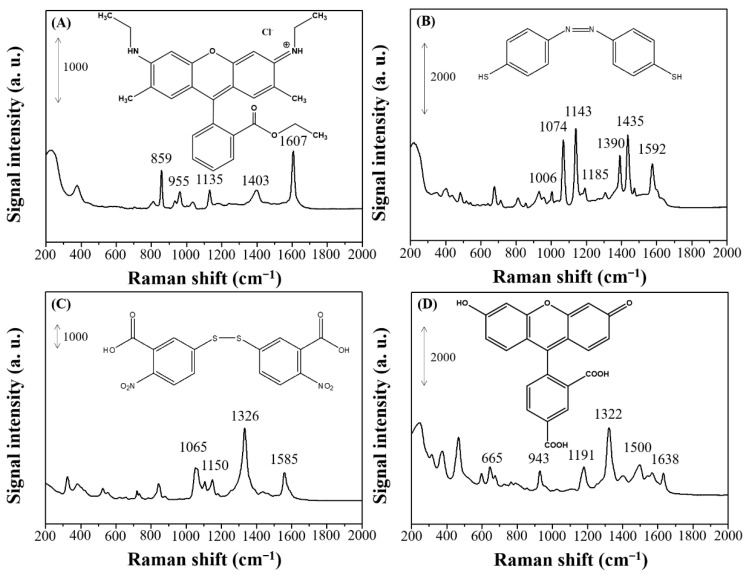
SERS spectra of (**A**) R6G, (**B**) 4-ATP, (**C**) DTNB, and (**D**) FAM molecules for the dendritic forest-like Ag nanostructures.

Common Raman reporters for SERS sensors were tested using the new Ag nanostructures. Figure 11 displays the SERS spectra of R6G, 4-ATP, DTNB, and FAM molecules on the dendritic forest-like Ag nanostructures. Figure 11A represents the SERS spectrum of R6G, which has characteristic Raman peaks at 859, 955, 1135, 1403, and 1607 cm^−1^. The strong peak at 1607 cm^−1^ was assigned to C–C stretching, whereas that at 1135 cm^−1^ was assigned to C–N stretching. According to literature, it is possible to occur photochemical conversion of 4-ATP into 4,4′-dimercaptoazobenzene (DMAB) on the Au or Ag surface [19]. Figure 11B finds the SERS spectrum of DMAB, and the Raman peaks at 1074 and 1592 cm^−1^ were attributed to ring breathing coupled with C–S and C–C stretching, respectively. The Raman peaks at 1143 and 1187 cm^−1^ were attributed to C–N stretching and C–H bending, respectively. Furthermore, the Raman peaks at 1390 and 1435 cm^−1^ were attributed to N–N and C–C stretching, respectively. Figure 11C shows the Raman spectrum of DTNB. The peaks at 1326 and 1585 cm^−1^ were assigned to the –NO_2_ and aromatic group stretching. Figure 11D represents the Raman spectrum of FAM. The strong peak at 1322 cm^−1^ was attributed to symmetric stretching of the hydroxyl group. In addition, some Raman peaks of the four analytes in Figure 11 does not provide vibration information and they could therefore be safely omitted. A linear relationships were obtained in the plots of Raman intensity versus the concentration of R6G, 4-ATP, DTNB, and FAM in the ranges of 2.0–20 nM (R^2^ = 0.9992), 0.6–1.4 µM (R^2^ = 0.9960), 1–100 µM (R^2^ = 0.9820), and 0.1–1.0 µM (R^2^ = 0.9560), respectively. Different linear ranges for different Raman reports were found due to different Raman cross section and chemical enhancement effect.

**Figure 12:**
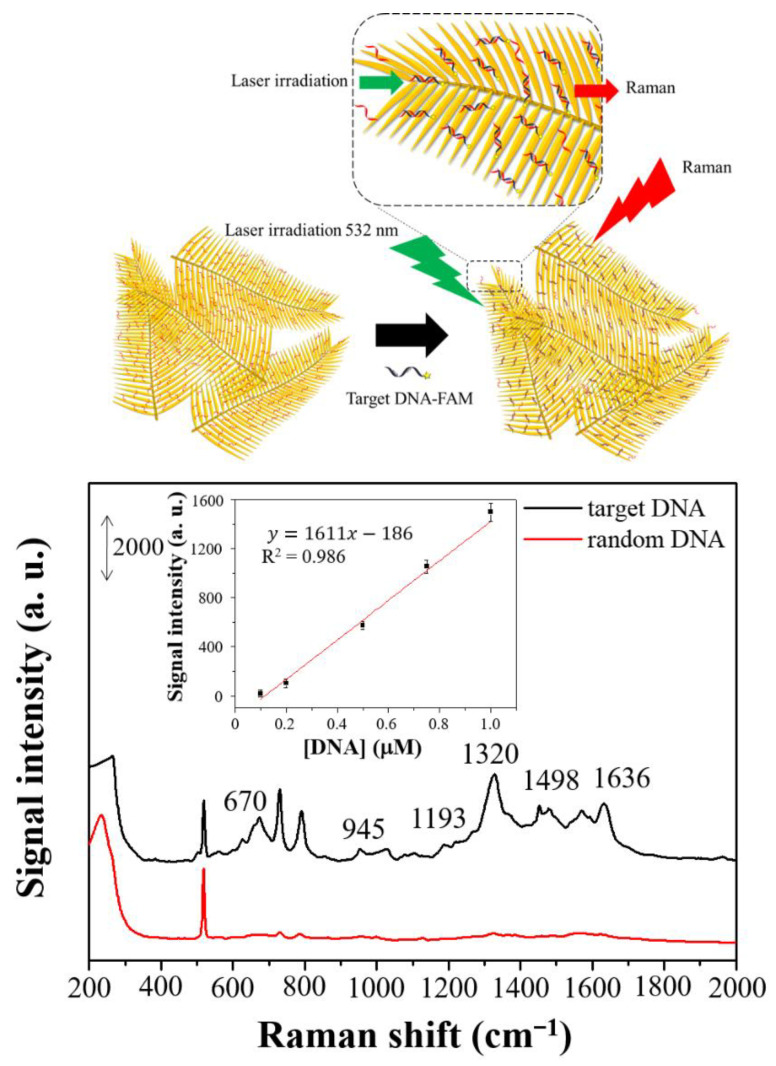
A cartoon representation of DNA sensing: Schematic of DNA sensing by the dendritic forest-like Ag nanostructures. SERS spectra of FAM obtained for a target single-stranded DNA (1.0 µM, black line) and random single-stranded DNA (1 µM, red line) attached to the dendritic forest-like Ag nanostructures. Linear responses (inset) of the Raman intensity plotted with respect to the concentration of target DNA. The error bars represent the standard deviations by using three batches of Ag substrates for which the average signal of 10 spots per sample are recorded.

The application of dendritic forest-like Ag nanostructures in biochemical detection based on DNA-hybridization mechanism was explored through detection and quantification of single-stranded DNA. First, complementary single strand DNA at the 3′ end was modified with thiol functional group, which was used to combine with the dendritic forest-like Ag nanostructures through Ag–S bonding. At the same time, target single strand DNA at the 5′ end was labeled with FAM as Raman report. Once target DNA and complementary DNA strand occurred hybridization, Raman signal could be found after water washing process. Figure 12 presents a cartoon representation of DNA sensing by the dendritic forest-like Ag nanostructures. A 3′ end thiol-terminated DNA strand (5′-CCAGATACTCACCGG-SH-3′), which can hybrid the fumarylacetoacetate hydrolase (*FAH*) gene, was used as the probe. This gene is mutated from the human genetic disease (hereditary tyrosinemia type 1). The black-line spectrum in Figure 12 reveals that the newly developed SERS detection system was sensitive to this target gene with an LOD of 33.5 ± 4.5 nM at signal-to-noise ratio of 3.0 (*n* = 3 and RSD% = 13.4%). LOD calculation was defined as 3.0 s/m (s being as the standard deviation calculated from the blank sample and m being as the slope of the calibration plot). The different positions in Raman peak of FAM molecules in Figure 11D and Figure 12 were found. It maybe because different orientations of FAM molecules were adsorbed on the surface of the prepared Ag substrates. In Figure 11D, free FAM molecules were randomly adsorbed on the Ag surface. However, FAM molecules were modified on 5′ end of DNA strand for DNA sensing. After hybridization. specific orientation of FAM-DNA strand was approached to the Ag surface. The details in specific orientation near to Ag substrate will be studied in the future. The Raman signal at 1320 cm^−1^ was linear with respect to the target DNA strand concentration over the range 0.1 to 1.0 µM (R^2^ = 0.986; inset in Figure 12). A featureless spectrum was obtained when a FAM-modified random target DNA strand was used (red-line spectrum in Figure 12). Thus, the dendritic forest-like Ag nanostructures detected DNA effectively.

## 4. Conclusions

In this study, a one-step FAGRR chemical approach was employed to deposit SERS-active dendritic forest-like Ag nanostructures on silicon wafers. The surface of roughness was due to the sharp positions and/or interstitial sites of the dendritic forest-like Ag nanostructures. The surface area was also increased because of interlacing between the dendritic forest-like Ag nanostructures. As a result, the prepared substrates produced a high EF value (9.18 × 10^8^). The FAGRR approach used for the preparation SERS substrates has the following excellent features compared with previously reported study (Table 2): (1) inexpensive and simple method: costly enzymes, complicated preparation, and sophisticated photolithographic techniques are not required; (2) short preparation time: the FAGRR approach requires only 1.0 min; (3) reliable and reproducible signal: the RSD% is lower than 0.617%; and (4) practicality: high-quality SERS spectra can be obtained for various Raman reporters used in SERS bio-assay (4-MBA, R6G, DMAB, DTNB, and FAM). As highly SERS-active substrates are continually being developed for improving the sensitivity of SERS-based bioassays, the straightforward method showed in this study can lead to the mass production of reproducible SERS-active substrates. Additionally, the dendritic forest-like Ag nanostructures deposited on the Si wafer can be combined with microelectromechanical systems and micro total analysis systems for label-free chemical and biological detection.

## Figures and Tables

**Table 1:** Experimental Raman shift (cm^−1^) of 4-MBA on the dendritic forest-like Ag nanostructures.

Raman Shift (cm^−1^)	Assignment ^a^
1584	9a
1476	15
1380	βO-H & υC-ph & 19a & *as.*υCO_2_
1180	βO–H & 9a
1135	9a & γO–H
1074	1 & υC–S
1012	υC–O & 1
932	17a
840	10b
771	6a & υC-ph & υC–O
713	11 & ωCO_2_
681	βCO_2_ & 19a & υC–S

^a^ The Wilson notation is employed. *as.*, asymmetry; υ, stretching vibration; β, bending vibration; γ, rocking vibration, ω, wagging vibration.

**Table 2:** Fabrication method, EFs, and applications of various flower-like Ag substrates.

Substrate	Preparation Process	EF	Applications	Ref.
Desert-rose-like Ag mesoparticles	One-step electrochemical reduction	1.3 × 10^5^ for 4-MBA	*FAH* gene	[18]
Silver dendrites	A facile in situ reduction method	7.0 × 10^5^ for 4-MBA	^a^	[26]
Ag nanodesert rose	Galvanic displacement process (one-step method)	2.0 × 10^10^ for R6G	^a^	[32]
Flower-like Ag microstructures	Chemical reduction on polyaniline/polyvinyl alcohol composite film (two-step method)	1.9 × 10^5^ for 4-MBA	^a^	[28]
Flower-like Ag-Au hetero-nanoparticles	Electrodeposition and galvanic replacement reaction	8.6 × 10^7^ for 4-MBA	^a^	[29]
Flower-like Silver Nanoparticles	Chemical reduction in the presence of ascorbic acid	10^7^ ~ 10^8^ for malachite green isothiocyanate	^a^	[33]
Flower-like Ag structures with concave surfaces	Electrodeposition	2.1 × 10^9^ for 4-ATP	^a^	[34]
Flower-like Ag nanostructures	Chemical reduction in the presence of _L_-cysteine	^a^	^a^	[35]
Flower-like Ag microstructures	Chemical reduction in the presence of surfactant	1.84 × 10^14^ for crystal violet	^a^	[36]
Dendritic forest-like Ag nanostructures	FAGRR process	9.18 × 10^8^ for 4-MBA	*FAH* gene	This study

^a^ Not provided.

**Table 3:** Reproducibility test for dendritic forest-like Ag nanostructures deposited on a Si wafer.

Raman Shift	1074 (cm^−1^)		1584 (cm^−1^)	
Day	Raman Intensity	RSD (%) ^a^	Raman Intensity	RSD (%)
Day 1	11,893 ± 25	0.210	19,909 ± 26	0.130
Day 2	11,747 ± 53	0.451	19,509 ± 95	0.487
Day 3	11,828 ± 73	0.617	19,588 ± 97	0.495

^a^ RSD is calculated by dividing the standard deviation by the mean value of the data set by using three batches of Ag substrates for which the mean value of 10 spots per sample are recorded.

## Data Availability

No applicable.
